# A Phase I/II Study Targeting Angiogenesis Using Bevacizumab Combined with Chemotherapy and a Histone Deacetylase Inhibitor (Valproic Acid) in Advanced Sarcomas

**DOI:** 10.3390/cancers10020053

**Published:** 2018-02-17

**Authors:** Varun Monga, Umang Swami, Munir Tanas, Aaron Bossler, Sarah L. Mott, Brian J. Smith, Mohammed Milhem

**Affiliations:** 1 Division of Hematology, Oncology, and Blood and Marrow Transplantation, University of Iowa Carver College of Medicine, Iowa City, IA 52242, USA; umang-swami@uiowa.edu (U.S.); mohammed-milhem@uiowa.edu (M.M.); 2 Department of Pathology, University of Iowa Carver College of Medicine, Iowa City, IA 52242, USA; munir-tanas@uiowa.edu (M.T.); aaron-bossler@uiowa.edu (A.B.); 3 Holden Comprehensive Cancer Center, Iowa City, IA 52242, USA; sarah-mott@uiowa.edu; 4 Department of Biostatistics, University of Iowa College of Public Health & Holden Comprehensive Cancer Center, Iowa City, IA 52242, USA; brian-j-smith@uiowa.edu

**Keywords:** sarcoma, valproic acid, rheostat, angiogenesis, epigenetics, bevacizumab

## Abstract

Epigenetic events and genetic alterations under the control of the tumor microenvironment potentially mediate tumor induced angiogenesis involved in soft tissue sarcoma (STS) metastasis. Addition of antiangiogenic agent, such as bevacizumab, to standard chemotherapy in treatment of sarcoma has been studied in clinical trials, but most of the findings have not supported its use. We hypothesized the existence of an epigenetically mediated “angiogenic switch”, and the tumor microenvironment, prevents bevacizumab from truly blocking angiogenesis. The addition of valproic acid (VPA), a weak histone deacetylase inhibitor, and bevacizumab, a monoclonal antibody against vascular endothelial growth factor, together with the cytotoxic effects of gemcitabine and docetaxel, may enhance responses and alter chemoresistance. This was designed as a phase I/II trial with primary endpoints including safety of the treatment combination and tumor response. Unresectable or metastatic sarcoma patients >18 years of age, irrespective of number of prior treatments, received VPA 40 mg/kg orally for 5 days prior to day 1, bevacizumab at 15 mg/kg IV on day 1, gemcitabine 900 mg/m^2^ (day 1, day 8), and docetaxel 75 mg/m^2^ (day 8). Cycles were of 28 day duration. Bevacizumab and VPA were continued as maintenance after 6 cycles, until disease progression. A standard 3 + 3 phase I dose de-escalation design was utilized to evaluate safety. Gain of function p53 gene mutation testing was performed on available archival tissue specimens. A total of 46 patients (30 female, 16 male) with median age of 60 (range 24–81) years were enrolled; 34 (73.9%) patients received prior chemotherapy, 14 (30%) of which received prior gemcitabine and docetaxel. Patients received a median of 5.5 cycles (range 0–24 of treatment (min 0, one patient died prior to completing the first cycle; max: 24, one patient received 6 cycles and 18 maintenance cycles before progressing). Seventeen patients underwent dose reduction, of which VPA was reduced in 6 patients. Forty-one patients were evaluable for response. There was a confirmed complete response in 1 (epithelioid sarcoma), and a partial response (PR) in 6 (1 carcinosarcoma, 2 extrauterine leiomyosarcoma (LMS), 2 undifferentiated pleomorphic sarcoma, and 1 uterine LMS) patients. Stable disease (SD) was seen in 21 patients for at least 2 months. One subject with prior gemcitabine and docetaxel had PR, and 7 had SD. Median progression-free survival (PFS) was 5.7 months (95% CI: 2.1–8.0), and overall survival (OS) was 12.9 months (95% CI: 8.3–14.5). Three patients died due to tumor progression while on the study. The combination of VPA, bevacizumab, gemcitabine, and docetaxel appears to be moderately safe and well tolerated. Given that there are very limited options for patients with relapsed refractory STS, this drug combination may be an important therapy to consider. This combination treatment deserves further investigation in epithelioid and carcinosarcoma subtypes.

## 1. Introduction

Soft tissue sarcomas (STSs) are a rare group of malignant tumors of various connective tissues originating primarily from the mesoderm. Approximately 12,000 new cases of sarcoma are diagnosed annually, representing about 1% of all cancer types, and 5000 will unfortunately succumb to this cancer [1]. Surgery with or without radiation therapy is the standard of care for patients with localized sarcoma. Chemotherapy is associated with response rates of 10–30% in metastatic STS at the expense of increased toxicity and a median survival of 12–18 months in total. Novel treatment approaches are desperately needed to help improve survival in these patients.

Neo-angiogenesis plays an important role in tumor metastases [2]. Vascular endothelial growth factor (VEGF) molecule has emerged as the key stimulus promoting angiogenesis in several malignancies, including sarcomas [3,4]. Relatively fewer clinical trials have evaluated the role of antiangiogenic therapy in sarcomas, despite strong preclinical data supporting a role for angiogenesis inhibition [5,6,7]. Bevacizumab is a recombinant human monoclonal antibody that binds human VEGF, and has been the most explored agent in various malignancies. It has had the greatest impact in improving overall survival in patients with metastatic colorectal cancer [8], but also has indication in metastatic non-small cell non squamous lung cancer [9], recurrent glioblastoma [10], and in advanced renal cancer [11]. The combination of gemcitabine, docetaxel, and bevacizumab was studied in patients with advanced or recurrent STS, and was deemed to be safe. Response rates of up to 31% were reported in chemotherapy naïve patients, with median response duration of 6 months [6]. Single agent bevacizumab showed a 13% response rate in patients with advanced or metastatic angiosarcoma and epithelioid hemangioendothelioma [7] (the vascular sarcomas).

Epigenetic manipulation is a novel approach to cancer therapy that is increasingly being explored in solid tumors with limited success. Clinical evidence supports that epigenetic silencing of the tumor suppressor genes with angiogenesis inhibiting properties may contribute to chemoresistance, and that drugs targeting epigenetic mechanisms may enhance chemosensitivity [12,13]. Mechanisms involving deregulation of acetylation and deacetylation play a causative role in the abnormal regulation of gene expression in many cancers [14,15,16]. Histone deacetylase inhibitors (HDACi) have a broad spectrum of antitumor effects, which includes downregulating VEGF [17] and suppressing neovascularization through alteration of other genes directly involved in angiogenesis and maintenance of tumor microenvironment [18,19,20]. Valproic acid (VPA) is a commonly prescribed antiepileptic drug with a well-established pharmacokinetic and toxicity profile [21]. It is known to have HDACi properties, and has shown antitumor effects [22]. Both pre-clinical [23] and clinical studies [22] have shown improved responses and reversal of resistance to cytotoxic drugs when HDACi were given as pretreatment with chemotherapeutic agents in various solid tumors. Histone modifying drugs and their metabolites can potentially modulate gene expression, and do so by acting as rheostats. We wanted to further explore this phenomenon. Gemcitabine, in combination with docetaxel, has shown superior survival in comparison to gemcitabine alone in STS [24]. The rationale for this trial was to potentially modify the tumor sensitivity to anti-angiogenesis and facilitate the cytotoxic effects of chemotherapy. The aim of this phase I/II study was to test the safety and efficacy of bevacizumab and histone deacetylase inhibitor VPA, in combination with gemcitabine and docetaxel, for treatment of metastatic STS, and to identify possible subgroups in sarcoma that may benefit from this approach and allow for proof of principle for use of this combination.

## 2. Results

### 2.1. Patient Characteristics and Disposition

**Figure 1:**
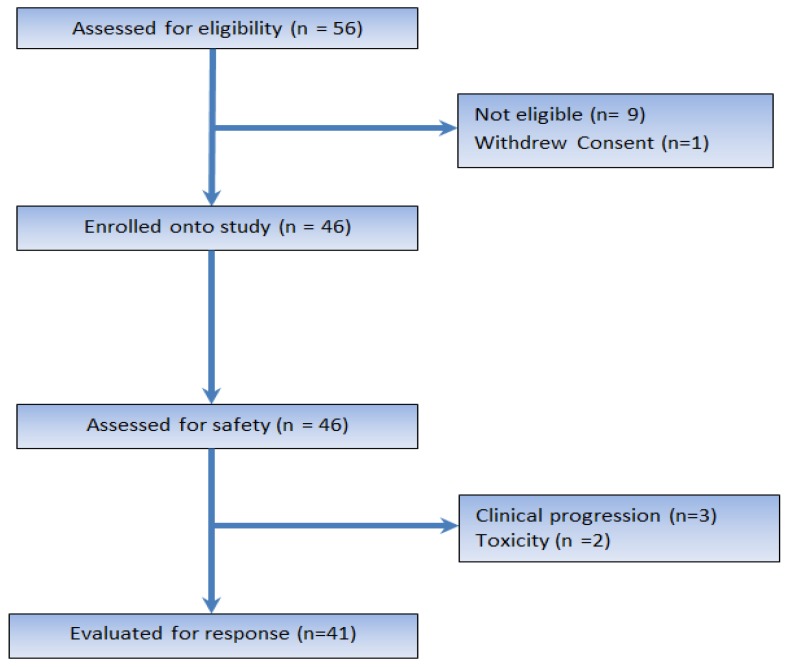
This is a CONSORT diagram showing the eligible, excluded and response-evaluable patients.

The study was conducted at Holden Comprehensive Cancer Center between 2008 and 2016. Patient disposition is listed in Table 1 and depicted in the flow diagram of Figure 1. Notably, 46 patients were enrolled in the study. Five patients came off the study prior to first disease assessment, either due to clinical progression or toxicity. Thirty patients (65.2%) were females and 16 patients (34.8%) were males. Leiomyosarcomas, which generally constitute 20 to 30% of all STSs, formed the biggest group in our study, including 16 patients (19.6% extra uterine leiomyosarcomas and 17.4% uterine leiomyosarcomas). Thirty-four (73.9%) patients had received chemotherapy prior to enrolling in this study; 14 patients (30.4%) had prior gemcitabine and docetaxel, either as adjuvant or metastatic disease treatment.

### 2.2. Safety and Toxicity

Safety cohort included patients who received a median of 5.5 cycles (range 0–24) of treatment. Two of the 6 patients initially enrolled to dosing cohort 1 of the phase I study portion were noted to develop grade 3 asymptomatic hyponatremia which was reversible. Both of those patients were on a low salt intake diet for their preexisting hypertension. No dose limiting toxicities were observed in dosing cohort 1. Thus, the study proceeded into phase II. Overall, 17 patients underwent dose reduction, of which VPA was reduced in 6 patients. Progression of disease lead to death in 3 patients while on study. Liver enzyme elevation and neurotoxicity attributable to valproic acid and hypertension attributed to bevacizumab were the most common toxicities (Table 2) apart from cytopenias from gemcitabine and docetaxel chemotherapy. 

### 2.3. Efficacy of Therapy

**Figure 2:**
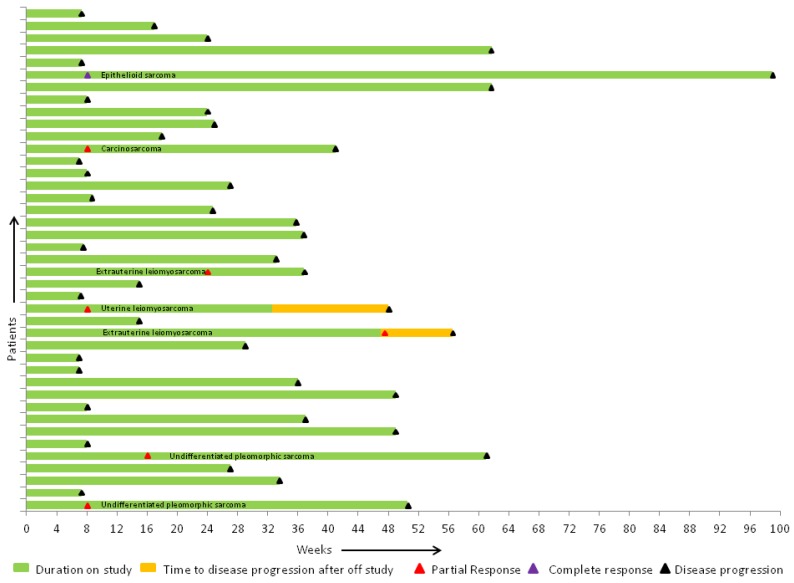
Summary of efficacy represented by swimmer’s plot. One complete response in epithelioid sarcoma and 6 partial responses in carcinosarcoma (1), extrauterine (1) and uterine leiomyosarcoma (2), and undifferentiated pleomorphic sarcoma (2).

**Figure 3:**
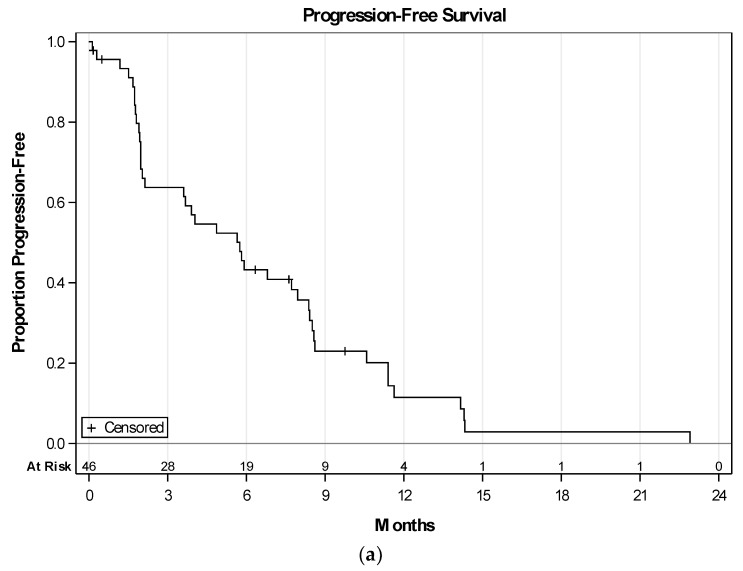

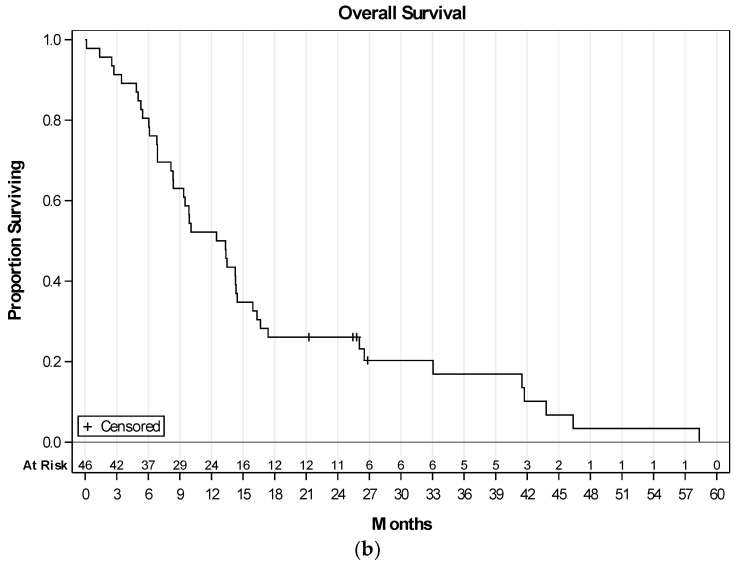
(**a**) Progression-free survival (PFS) in 46 treated patients. Median PFS: 5.7 months (95% CI: 2.1–8.0). PFS was calculated from date of study treatment initiation to progression or death from any cause. Patients removed due to toxicity were censored at date of removal; (**b**) overall survival (OS) in 46 treated patients. Median OS: 12.9 months (95% CI: 8.3–14.5). Patients removed due to toxicity were censored at date of removal.

Of the 41 evaluable patients who received at least 2 cycles of per protocol therapy and where response was evaluable, there was a complete response in 1 (epithelioid), and a partial response (PR) in 6 (1 carcinosarcoma, 2 extrauterine LMS, 2 undifferentiated pleomorphic, and 1 uterine LMS) patients (Figure 2). Stable disease (SD) was seen in 21 patients. One subject with prior gemcitabine and docetaxel had PR, and 7 had SD (Table 3). Of the 46 patients enrolled, median progression-free survival (PFS) was 5.7 months (95% CI: 2.1–8.0), and overall survival (OS) was 12.9 months (95% CI: 8.3–14.5) (Figure 3a,b). In the LMS subgroup, median PFS was 8.4 months (95% CI: 2–8.6), and median OS was 16.3 months (95% CI: 8.1–26.5). Addition of VPA and bevacizumab, to gemcitabine and docetaxel, showed a clinical benefit rate of 61.5% (8/13) in patients with prior gemcitabine and docetaxel exposure, either as neoadjuvant or adjuvant therapy. Mean level of serum VPA level was available for 41 patients, and was 115 microgram/mL. Gain of function (GOF) *TP53* mutations were found in 3 and loss of function (LOF) *TP53* in 5 of the 30 available tumor specimens.

## 3. Discussion

The mainstay of treatment of metastatic STS is resection outside of removing the primary tumor. Growth of sarcomas is, in part, dependent on neoangiogenesis. We attempted to show that epigenetically modifying the tumor microenvironment may turn off the “angiogenic switch”, improve cytotoxic cell kill, overcome chemotherapy resistance, and thereby, improve response rates. Participation in a clinical trial remains the standard of care treatment of metastatic STS. Here we explored the combination of weak histone deacetylase inhibitor VPA, together with angiogenic inhibitor bevacizumab, in combination with standard chemotherapy gemcitabine and docetaxel, in a single arm phase I/II trial.

VPA is a short chain fatty acid, and has a well-established toxicity profile because of its use as antiepileptic agent. VPA is also a relatively inexpensive drug, and overall, well tolerated. It is also known to have antiangiogenic activity, and has weak histone deacetylase (class I and II) activity. Pretreatment with histone deacetylase inhibitors in prior clinical studies [22,25] has shown improved response rates to chemotherapy. There was no dose limiting toxicity or maximum tolerated dose related to VPA reported in our study. The precise dose level of VPA in serum at which histone deacetylase activity is at the peak, is not known. While we did not document VPA level prior to every dose of chemotherapy, we attempted to achieve >85 mcg/mL which has been shown in studies to be associated with decreased histone acetylase activity [22]. As VPA gets removed from the microenvironment, it does so in the presence of chemotherapy. This variability in VPA concentration in the tumor tissue could potentially have had a rheostat effect in tweaking the gene expression, and perhaps making the chemotherapy more effective. Histone acetylation assays were not measured as this is not a reliable marker, and is a significant limitation of this study.

Bevacizumab has shown to significantly impact overall survival in patients with colon cancer in a pivotal phase 3 trial, although the increases in response rates were modest, from 35% to 45% [8]. It has been tested in four different clinical trials, either as single agent or combination with chemotherapy in STS, and showed a modest activity in some histological subtypes, such as angiosarcomas, epithelioid hemangioendotheliomas, and undifferentiated sarcomas [5,6,7,26]. We used 15 mg/kg every 4 weeks for dosing of bevacizumab, for scheduling convenience. This dosing strategy could potentially have contributed to the rheostat effect.

Like many tumors, *TP53* mutations have been reported in STS [27,28,29]. The functional impact of different types of p53 mutant proteins may have different implications for chemosensitivity. Some variants are relatively inconsequential from the perspective of p53 function, and proteins of this type retain wild type activity. Other mutations are LOF or p53-null, in which single amino acid changes completely inactivate or destabilize the protein. Preclinical studies have shown that LOF p53 mutant sarcomas produced significantly more VEGF, which contribute directly to angiogenesis, metastasis, and growth [30]. Recent retrospective data suggests that LOF *TP53* mutant sarcomas may be more sensitive to VEGF inhibitors [31]. Finally, an interesting category of *TP53* mutations is the GOF or “oncogenic” *TP53* mutations, that convert p53 from a tumor suppressor to an oncogene, also termed as oncomorphic p53, as proposed by Brachova et al. [32]. Treatment with HDAC inhibitor of ovarian cancer *TP53* GOF mutant cell lines lead to potential dissociation of GOF p53–Hsp 90 complex, thereby leading to mutant p53 degradation. We evaluated the available archival tumor blocks for gain of function *TP53* mutations, but insufficient numbers of variants were detected to meet statistical significance (Table 4). This perhaps reflects either the dynamic nature of these mutations, or the use of weak HDAC inhibitor in our study, and a failure to achieve the rheostat effect.

The addition of VPA to bevacizumab was beneficial for several reasons. Sixty-one percent of patients who had failed gemcitabine/docetaxel alone, responded (complete or partial) to the 2 drug addition. This clinical benefit speaks to better understanding what mechanism(s) is (are) involved. Furthermore, therapeutically, the addition of these two agents should be considered in patients whose disease progresses after 2 cycles of gemcitabine/docetaxel. This pilot study reveals the possible addition of drugs that affect tumor microenvironment to the chemotherapies that affect cell division, and push towards repurposing of older agents. VPA as a HDAC inhibitor has a weaker activity, and agents with more potent HDAC inhibitory activity, such as panobinostat and vorinostat, are now available. A combination of these newer HDAC inhibitors with pazopanib, an agent with anti-VEGF activity, could potentially be explored in metastatic STS.

## 4. Materials and Methods

### 4.1. Patient Eligibility

Eligible patients had histologically confirmed metastatic STS with measurable disease, as defined by RECIST 1.0, with documented disease progression on their most recent prior therapy. Inclusion criteria included age ≥18 years, recovery from prior radiation or chemotherapy side effects, ECOG performance status of ≤2, and ability to provide informed consent. Any number of prior lines of therapy including untreated patients was allowed. Patients who have received prior anthracycline underwent a baseline echocardiogram or MUGA scan with LVEF ≥ the lower limit of institutional normal. Screening EKG with a QTc less than 450 msec (Bazett’s formula) was performed prior to enrollment. Prior exposure to gemcitabine and taxotere was allowed if residual toxicity from previous treatments was less than or equal to grade 1. All subtypes of STS, except Kaposi’s sarcoma and gastrointestinal stromal tumor, were included.

Patients with history of prior use of bevacizumab, HDACi, heat shock protein 90 inhibitors, or VPA treatment of cancer or any other condition, inadequately controlled hypertension as defined by systolic blood pressure ≥150 mm·Hg or diastolic blood pressure ≥100 mm·Hg, prior history of hypertensive crises or hypertensive encephalopathy, history of myocardial infarction, unstable angina, stroke or transient ischemic attack, bowel perforation within 6 months prior to day 1 of therapy, treated brain metastases within 3 months prior to day 1, history of hemoptysis within 1 month prior to day 1, evidence of bleeding diathesis, non-healing ulcer or wound, urine protein creatinine ratio ≥1.0 at screening, major surgical procedure within 28 days prior to day 1 or history of any other malignancy (except for curatively treated carcinoma in situ of cervix, basal or squamous cell carcinoma of the skin) within 2 years, and pregnant women, were all excluded.

Written informed consent was required. The study was approved by the institutional review board, and conducted in accordance with the US FDA Good Clinical Practice Requirements. The trial was registered at clinicaltrials.gov (NCT01106872).

### 4.2. Therapy

Patients were treated with pulsed dosing of oral VPA at 40 mg/kg (dose 0) per day for 5 days prior to day 1 of the cycle, and were repeated before each cycle. Patients received gemcitabine 900 mg/m^2^ intravenously (iv) over 90 min followed by bevacizumab 15 mg/kg iv, as per standard infusion guidelines on day 1. Gemcitabine 900 mg/m^2^ iv over 90 min followed by docetaxel 75 mg/m^2^ iv over 60 min were administered on day 8. Any grade 3 or 4 neutropenia on day 1 or day 8, or febrile neutropenia, required use of growth factor support, starting on day 9 or day 10 and to be continued in all subsequent cycles. Dexamethasone was used as premedication for docetaxel, starting day 7 and continuing through day 9. Each cycle was of 28 day duration (Supplementary Materials Figure S1). Up to 1 dose reduction in gemcitabine, docetaxel, and VPA was permitted for significant protocol-defined toxicities. No dose reductions were allowed for bevacizumab. Patients were treated for a total of 6 cycles, and assessed every 2 cycles for progression. For exceptional responders (continued partial or complete response at 6th cycle) more than 6 cycles of chemotherapy, if well tolerated, was permitted, as per investigator. Upon completion of 6 cycles of chemotherapy maintenance treatment with pulsed VPA and bevacizumab, treatment was continued until unacceptable toxicity or progression. VPA serum levels were obtained on day 1.

### 4.3. Study Parameters

Toxicities were assessed using the NIH-NCI Common Terminology Criteria for Adverse Events, version 3.0 (CTCAE v3.0). A computed tomography of chest, abdomen, and pelvis with intravenous contrast was required within 4 weeks of starting therapy, and was repeated every 8 weeks to determine response to treatment. Disease progression and best response to study treatment were determined as per RECIST 1.0 criteria.

### 4.4. Analysis Samples

All pathological diagnoses were confirmed by a pathologist with subspecialty training in bone and soft tissue pathology. *TP53* mutation testing was performed on DNA from available archival diagnostic tissue specimens using next generation sequencing by synthesis platform (ThermoFisher Scientific, Waltham, MA, USA).

### 4.5. Statistical Considerations and Analysis

A standard 3 + 3 dose de-escalation design with two VPA dosing cohorts: (1) 40 mg/kg dose, and (2) 20 mg/kg dose, was employed in the phase 1 portion of the study. Patients were initially enrolled to dosing cohort 1—the anticipated dose for the subsequent phase II portion—to evaluate efficacy of the proposed treatment regimen with respect to a selection of tumor subtypes.

Descriptive statistics are reported to summarize patient and clinicopathologic characteristics. Adverse events were tabulated by type and grade for all patients who received at least one study treatment. Treatment efficacy was evaluated by tumor response, progression-free survival (PFS), and overall survival (OS). Time was calculated from treatment initiation to progressive disease or death due to any cause for PFS, or death due to any cause for OS. Patients removed from the trial due to toxicity were censored. Survival probabilities were estimated and plotted using the Kaplan–Meier method. Survival estimates are reported along with 95% confidence intervals. Summaries and plots were derived using SAS v9.4 (SAS Institute, Cary, NC, USA).

## 5. Conclusions

The combination of VPA, bevacizumab, gemcitabine, and docetaxel appears to be moderately safe. Given the limited treatment options for patients with relapsed refractory STS, this drug combination could potentially be considered. Interesting responses were seen in patients with epithelioid sarcoma and carcinosarcoma subtypes. More potent HDAC inhibitor should be explored in future epigenetic studies.

## Figures and Tables

**Table 1:** Baseline patient characteristics and patient disposition.

Characteristic	Level	*N* (%)
Gender	Female	30 (65.2)
	Male	16 (34.8)
Tumor Histology	Angiosarcoma	4 (8.7)
	Carcinosarcoma	4 (8.7)
	Epithelioid Sarcoma	2 (4.3)
	Extrauterine leiomyosarcoma	9 (19.6)
	Undifferentiated uterine sarcoma	2 (4.3)
	Liposarcoma	4 (8.7)
	MPNST ^1^	2 (4.3)
	Rhabdomyosarcoma	1 (2.2)
	Malignant solitary fibrous tumor	1 (2.2)
	Synovial sarcoma	2 (4.3)
	Undifferentiated Pleomorphic sarcoma	7 (15.2)
	Uterine Leiomyosarcoma	8 (17.4)
^2^ ECOG	0	21 (45.7)
	1	24 (52.2)
	2	1 (2.2)
Prior chemotherapy	No	12 (26.1)
	Yes	34 (73.9)
Prior lines of chemotherapy	1	15 (32.6)
	2	8 (17.4)
	3	8 (17.4)
	4	1 (2.2)
	5	1 (2.2)
	6	1 (2.2)
Prior Gemcitabine + Docetaxel	No	32 (69.6)
	Yes	14 (30.4)
Prior radiation	No	30 (65.2)
	Yes	16 (34.8)
Prior surgery	No	2 (4.3)
	Yes	44 (95.7)

^1^ MPNST, malignant peripheral nerve sheath tumor; ^2^ ECOG, Eastern Cooperative Oncology Group performance status.

**Table 2:** Adverse events possible, probable, or definitely attributable to bevacizumab or valproic acid, all cycles.

Toxicity	Grade				
	2	3	4	5	Total
Anemia	1	1	1	0	3
Leukopenia	1	1	1	0	3
Neutropenia	0	1	0	0	1
Thrombocytopenia	1	1	1	0	3
Hypertension	5	4	0	0	9
Elevated INR	0	1	0	0	1
Fatigue	5	0	0	0	5
Alopecia	3	0	0	0	3
Diarrhea	2	0	0	0	2
Heartburn	3	0	0	0	3
Mucositis	2	0	0	0	2
Nausea	2	0	0	0	2
Taste Alteration	1	0	0	0	1
Vomiting	1	0	0	0	1
Hemorrhage	2	1	0	0	3
Leg edema	1	0	0	0	1
Hyponatremia	0	7	0	0	7
Liver Enzyme Elevation	25	5	0	0	30
Confusion	2	2	0	0	4
Dizziness	2	1	0	0	3
Memory Impairment	1	0	0	0	1
Neurology Other	4	1	0	0	5
Psychosis-Hallucination	0	1	0	0	1
Somnolence	2	0	0	0	2
Oral pain	1	0	0	0	1
Headache	0	1	0	0	1
Myalgia	2	0	0	0	2
Voice Changes-Hoarseness	1	0	0	0	1
Voice Changes-Slurred Speech	1	0	0	0	1
Urinary incontinence	0	1	0	0	1
*Total*	*71*	*29*	*3*	*0*	*103*

INR: International Normalized Ratio.

**Table 3:** Clinical outcomes in 41 evaluable patients who received at least 2 cycles of protocol therapy. Data are given as No. (%).

		Best Response			
		CR *N* = 1	PR *N* = 6	SD *N* = 21	PD *N* = 13
Diagnosis	Angiosarcoma	0 (0)	0 (0)	3 (100.0)	0 (0)
	Carcinosarcoma	0 (0)	1 (33.3)	2 (66.7)	0 (0)
	Epithelioid sarcoma	1 (50)	0 (0)	1 (50)	0 (0)
	Extra Uterine Leiomyosarcoma	0 (0)	2 (22.2)	5 (55.6)	2 (22.2)
	High Grade/Undifferentiated Uterine Sarcoma	0 (0)	0 (0)	1 (50.0)	1 (50.0)
	Liposarcoma	0 (0)	0 (0)	0 (0)	4 (100.0)
	MPNST	0 (0)	0 (0)	0 (0)	1 (100.0)
	Rhabdomyosarcoma	0 (0)	0 (0)	1 (100.0)	0 (0)
	SFT	0 (0)	0 (0)	1 (100.0)	0 (0)
	Synovial	0 (0)	0 (0)	1 (50.0)	1 (50.0)
	Undifferentiated Pleomorphic/Spindle Cell Sarcoma	0 (0)	2 (40.0)	1 (20.0)	2 (40.0)
	Uterine Leiomyosarcoma	0 (0)	1 (12.5)	5 (62.5)	2 (25.0)
Leiomyosarcomas		0 (0)	3 (17.7)	10 (58.8)	4 (23.5)
Prior chemotherapy	No	1 (9.1)	2 (18.2)	6 (54.5)	2 (18.2)
	Yes	0 (0)	4 (12.9)	15 (48.4)	12 (38.7)
Prior Gemcitabine + Docetaxel	No	1 (3.4)	5 (17.2)	14 (48.3)	9 (31.0)
	Yes	0 (0)	1 (7.7)	7 (53.8)	5 (38.5)
Gain of function p53 mutation	No	1 (2.9)	5 (14.7)	17 (50.0)	11 (32.4)
	Yes	0 (0)	0 (0)	2 (66.7)	1 (33.3)

CR, complete response; PR, partial response; SD, stable disease; PD, progressive disease.

**Table 4:** Types of *TP53* mutations in different histologic subtypes of soft tissue sarcomas with their respective position and allele frequencies. * represents likely gain of function mutations

Histology	TP53 Mutation	Variant	Genomic Position	Allele Frequency
Pleomorphic Sarcoma	GOF	* exon7:c.G743A:p.R248Q [33] exon5:c.C451A:p.P151T	chr7: 7577538 chr7: 7578479	33% 31%
Uterine leiomyosarcoma	GOF	* exon5:c.G524A:p.R175H [33]	chr7: 7578406	90%
Retroperitoneal leiomyosarcoma	GOF	* exon8:c.844C > G; p.R282G [34,35]	chr7: 7577094	72%
Uterine leiomyosarcoma	LOF	exon6:c.C574T:p.Q192X	chr7: 7578275	84%
Uterine Leiomyosarcoma	LOF	exon10:c.C1024T:p.R342X	chr7: 7574003	95%
Carcinosarcoma	LOF	exon2:c.52delA:p.T18fs	chr7: 7579860	38%
Extra-uterine Leiomyosarcoma	LOF	exon4:c.205delG:p.A69fs	chr7: 7579481	56%
Extra-uterine leiomyosarcoma	LOF	exon8:c.785delG:p.G262fs	chr7: 7577152	59%

GOF, gain of function; LOF, loss of function.

## Supplementary Materials

The following are available online at http://www.mdpi.com/2072-6694/10/2/53/s1, Figure S1: Schematic representation of the treatment plan and different doses of drugs used.
