# Supplementary material for: A Phase I/II Study Targeting Angiogenesis Using Bevacizumab Combined with Chemotherapy and a Histone Deacetylase Inhibitor (Valproic Acid) in Advanced Sarcomas
Source: Cancers (Basel). 2018 Feb 17;10(2):53. doi: 10.3390/cancers10020053 (PMC5836085; doi:10.3390/cancers10020053)
Supplement: Supplementary file 1 [file cancers-10-00053-s001.pdf]

cancers

Supplementary Material

A Phase I/II Study Targeting Angiogenesis Using Bevacizumab Combined with Chemotherapy and a Histone Deacetylase Inhibitor (Valproic Acid) in Advanced Sarcomas

Varun Monga, Umang Swami, Munir Tanas, Aaron Bossler, Sarah L. Mott, Brian J. Smith and Mohammed Milhem

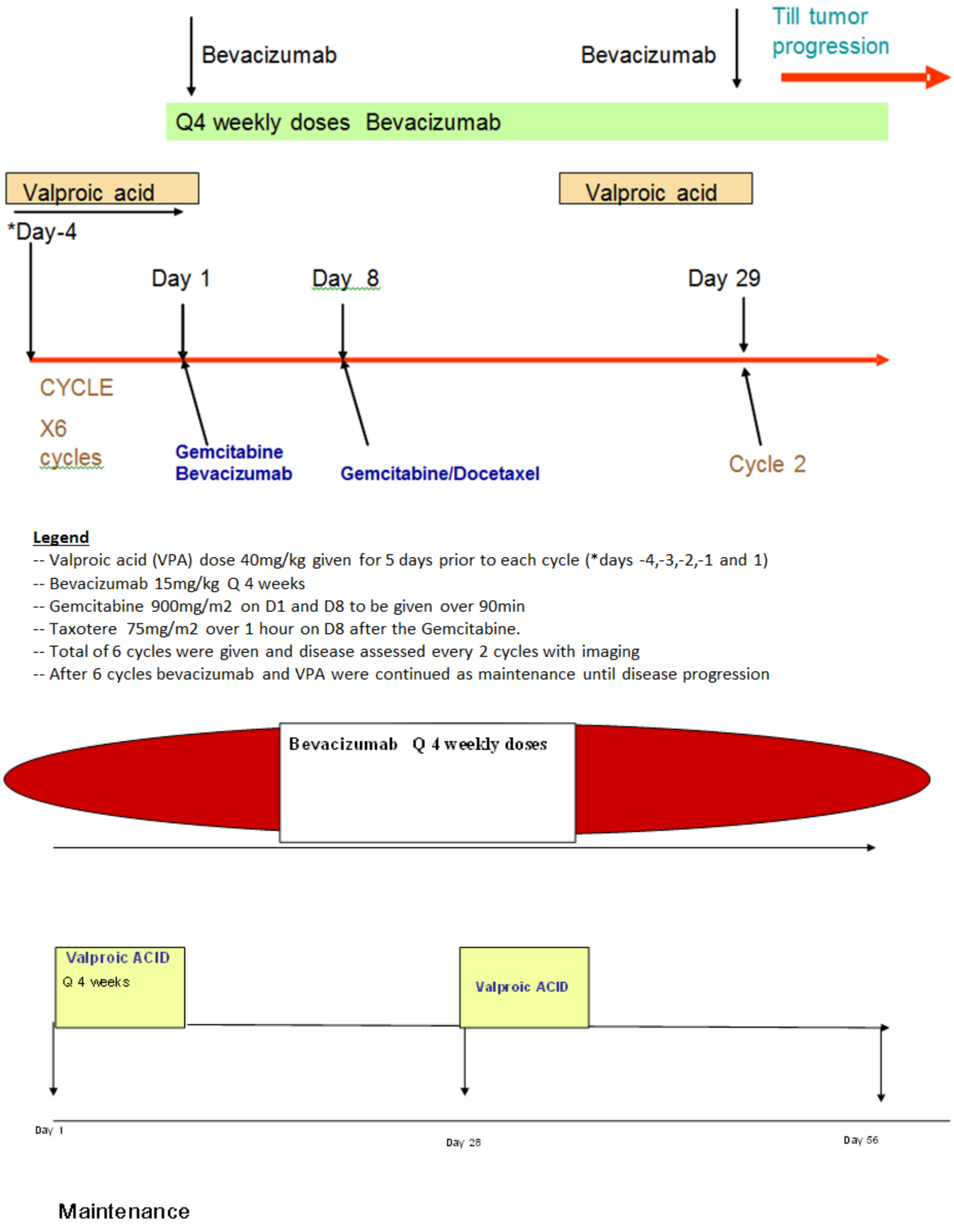

Figure S1. Schematic representation of the treatment plan and different doses of drugs used. During maintenance phase patients received Bevacizumab at 15mg/kg Q 4 weeks and Valproic acid at 40mg/kg q 4 weeks for 5 days until tumor progression.
